# Prevalence of non-communicable disease risk factors and their association with economic status: findings from the 2021 health behaviour of population survey in Thailand

**DOI:** 10.1080/16549716.2025.2485689

**Published:** 2025-05-22

**Authors:** Polathep Vichitkunakorn, Warintorn Bunyanukul, Kanarit Apiwan, Detphop Tanasanchonnakul, Monsicha Sittisombut

**Affiliations:** a Department of Family and Preventive Medicine, Faculty of Medicine, Prince of Songkla University, Hat Yai, Songkhla, Thailand; b School of Medicine and Health Sciences, Faculty of Medicine, Prince of Songkla University, Hat Yai, Songkhla, Thailand

**Keywords:** Lifestyle, behaviour risk factors, obesity, sedentary behaviour, socioeconomic factors

## Abstract

**Background:**

Non-communicable diseases (NCDs) are major contributors to mortality and disease burden; however, evidence regarding NCD risk factors, particularly socioeconomic factors, remains limited.

**Objectives:**

We investigated the prevalence of five key behavioural risk factors for NCDs (smoking, alcohol consumption, physical inactivity, unhealthy diet, and overweight/obesity) within the Thai population and the influence of economic status on these risk factors.

**Methods:**

We gathered secondary data from the 2021 health Behaviour of Population Survey. Data were derived from a stratified, nationally representative household survey using two-stage sampling. Economic status was categorised into very low, low, middle, high, and very high levels.

**Results:**

Among the 207,191 participants (weighted to represent 26,600,947 participants), the most prevalent NCD risk factor was an unhealthy diet (56.93%), followed by overweight/obesity (50.03%), physical inactivity (42.70%), alcohol consumption (29.73%), and smoking (16.61%). Higher economic levels were associated with increased odds of alcohol consumption (e.g. adjusted odds ratio [AOR] = 1.13, 95% CI: 1.03–1.25 for high) and an unhealthy diet (AOR = 1.26, 95% CI: 1.15–1.38 for very high), while smoking odds decreased (AOR = 0.67, 95% CI: 0.59–0.77 for very high). Physical inactivity exhibited a U-shaped association, and overweight/obesity slightly increased at the highest economic levels (AOR = 1.10, 95% CI: 1.01–1.21).

**Conclusion:**

Unhealthy dietary patterns and overweight/obesity were the most prevalent NCD risk factors. Smoking was the least prevalent. Tailored, evidence-based interventions targeting specific economic groups are needed to effectively reduce NCD risk factors and promote health equity.

## Paper Context

- **Main findings:** A higher economic status in Thailand was associated with increased odds of alcohol consumption and unhealthy diets but a lower prevalence of smoking; overweight or obesity was slightly more prevalent in the very high economic group, and physical inactivity demonstrated a U-shaped relationship across economic extremes.
- **Added knowledge:** To our knowledge, this study is the first to explore the prevalence and socio-demographic determinants of key risk factors for non-communicable diseases using nationally representative Thai data, highlighting the nuanced impact of economic levels on health behaviours.
- **Global health impact for policy and action:** Our findings emphasise the urgent need for targeted, equity-focused interventions and integrated policy measures to address socio-economic disparities in health behaviours and reduce the global burden of non-communicable diseases.

## Background

Non-communicable diseases (NCDs) are linked to modifiable lifestyle risk behaviours and progress slowly, gradually accumulating symptoms over time. NCDs are a leading health issue globally [1]. In Thailand, they are a major contributor to mortality and the overall disease burden, accounting for 76% of all deaths. Of these, 86% occur in participants aged below 70 years, and the World Health Organization (WHO) classifies these as premature deaths [2]. NCDs include cardiovascular disease, diabetes, cancer, and chronic obstructive pulmonary disease [1]. The number of participants at risk of developing NCDs is rapidly increasing and the increase is expected to continue. Furthermore, the growing burden of NCDs threatens the sustainability of Thailand’s universal health coverage system, with over 139 billion baht currently being spent on treating these diseases. The economic impact is also significant, amounting to over 1.5 trillion baht owing to premature deaths, frequent absenteeism, and reduced work capacity. Consequently, NCDs profoundly affect social and economic development as well as the long-term fiscal sustainability of the government [2].

Most NCDs are preventable as their increase is primarily due to five key risk behaviours: smoking; alcohol consumption; physical inactivity; an unhealthy diet (high in sugar, fat, and salt); and metabolic risk factors, such as hypertension, overweight or obesity, hyperglycaemia, and hyperlipidaemia. Reducing or modifying these risky behaviours can decrease the likelihood of developing NCDs by approximately 80%. Adoption of healthy behaviours such as quitting smoking, reducing alcohol consumption, exercising regularly, and reducing the intake of foods high in sugar, fat, and salt can significantly lower the risk of developing NCDs. Prevention of NCDs reduces the incidence of these diseases and improves the population’s overall quality of life and life expectancy. Studies in England [3], Brazil [4], and India [5] have revealed that the clustering of risk factors varies by population, with common co-occurring risk factors identified particularly among men and participants with a low economic status.

Economic status plays a significant role in the prevalence of behavioural risk factors for NCDs. Studies have shown that individuals with lower economic statuses are more likely to engage in risky behaviours, such as smoking, excessive alcohol consumption, and unhealthy eating habits. These behaviours often occur in clusters, leading to a high risk of developing NCDs [6,7]. Economic constraints may limit access to healthier food options, recreational facilities for physical activity, and healthcare services, further exacerbating this risk [6–9]. The socio-economic determinants of these risk behaviours are crucial for developing targeted interventions to reduce the burden of NCDs and promote health equity [7,8].

Despite extensive global research on NCD risk factors, evidence from Thailand remains limited, particularly regarding the influence of socio-economic factors. In this study, we address this gap by utilising data from the 2021 health Behaviour Survey conducted by the National Statistical Office (NSO), the first analysis of its kind using nationally representative Thai data. Thus, our primary objective was to estimate the prevalence of five key behavioural risk factors for NCDs – namely, smoking, alcohol consumption, physical inactivity, an unhealthy diet, and overweight or obesity – within the Thai population. The secondary objective was to examine the influence of socio-demographic and economic characteristics, particularly economic status, on these risk factors.

## Methods

### Study design and setting

We used data from the 2021 health Behaviour of Population Survey, conducted by the NSO of the Ministry of Digital Economy and Society, Thailand. The Health Behaviour of Population Survey is a nationally representative, cross-sectional household survey and is among the largest health surveys in Thailand. A stratified two-stage sampling design was used, with provinces as the strata, and within each province, urban and rural areas were substrata for the first sampling stage (primary sampling stage). The primary sampling units were the enumeration districts. In the second sampling stage (secondary sampling stage), the participating households were the secondary sampling units, with 16 sample households per enumeration district in urban and rural areas. This resulted in a sample of 84,000 households. We interviewed all members of each household. The final number of participants was 207,191.

### Data collection

In this study, we conducted a secondary analysis of the NSO data. The NSO ensured complete anonymity by excluding personally identifiable information such as names, addresses, or national identification numbers. We received the data electronically through a secure system accessible only to authorised users with access codes. The NSO collected data across the country between 1 February and 31 May 2021. Household representatives were interviewed using tablets instead of traditional paper questionnaires. This method facilitated real-time quality control and performance monitoring through web applications. The application also offers features for employee surveys and work progress tracking. Finally, after data verification for accuracy and completeness by the NSO staff, the anonymised data were securely transferred and stored in a cloud computing system.

### Dependent variable – behavioural risk factors for non-communicable diseases

We assessed the behavioural risk factors for NCDs using a self-administered questionnaire. According to WHO guidelines, NCDs have five key risk factors. Each behavioural risk factor was defined as follows (Table 1 and Table S1 in the Supplementary Appendix): smoking referred to current tobacco use (smoking, smokeless tobacco, or both) and carries health risks, with no safe level of tobacco use (no specific timeframe) [1,5]; alcohol consumption was assessed as current alcohol consumption, defined as consumption of any form of alcoholic beverage (including beer, brandy, and whiskey) within the past year [1,5]; unhealthy diet referred to unhealthy dietary habits, defined as consumption of high amounts of sugar, salt, or fat within the past month [10]; physical inactivity was characterised by engagement in insufficient physical activity to meet the recommended levels necessary for maintaining health [1,10]; and overweight or obesity was determined using a body mass index (BMI) cut-off of 23 kg/m^2^, which is recommended for Asian populations owing to their increased risk of NCDs at lower BMI levels compared with other populations [11]. This threshold aligns with the WHO expert consultation on BMI cut-offs for Asian populations that highlighted the increased risks of diabetes and cardiovascular diseases associated with low BMI ranges.

**Table 1.** Description of non-communicable disease risk factors analysed in the present study.

	Definition	
Risk factor	Yes	No
Smoking	Current tobacco use (no specific timeframe).	Never used tobacco (no specific timeframe).
Alcohol consumption	Current alcohol consumption within the past year.	Never drank alcoholic beverages and former occasional drinker, but not in the past year.
Unhealthy diet	Consuming unhealthy foods five or more days per week in the past month.	Never consuming unhealthy foods or consuming them fewer than five days per week in the past month.
Physical inactivity	Those aged 15–17 years engaging in less than 420 minutes of moderate or high-intensity activity per week or those aged 18 years and older engaging in less than 150 minutes of moderate-intensity activity or 75 minutes of high-intensity activity per week.	Those aged 15–17 years engaging in at least 420 minutes of moderate or high-intensity activity per week, or those aged 18 years and older engaging in at least 150 minutes of moderate-intensity activity or 75 minutes of high-intensity activity per week.
Overweight or obesity	Body mass index (BMI) ≥ 23 kg/m^2^	BMI < 23 kg/m^2^

### Independent variable – economic level

Economic status was categorised into five levels: very low (income less than 4,433.5 baht/month), low (income 4,433.5–6,959.5 baht/month), middle (income 6,959.5–10,262 baht/month), high (income 10,262–19,908 baht/month), and very high (income >19,908 baht/month) [12].

### Other independent variables

The following socio-demographic data were collected: sex, age, place of residence, education, living arrangements, economic status, and Thai region. The study included male and female participants aged 15 years or older, categorised into five age groups: 15–19, 20–24, 25–44, 45–59, and 60 years or older. The place of residence was classified as urban or rural. Educational attainment was categorised as no, primary, secondary, and higher education. Living arrangements were classified as never married, living with a partner, or not living with a partner. Participants were also grouped based on the five regions of Thailand: Bangkok and the central, northern, north-eastern, and southern regions.

### Statistical analysis

Sample weights were applied using the survey package in the R program to account for the survey design and ensure that the study population reflected national demographics. Frequencies and percentages were used to describe the distribution of participants based on socio-demographic and economic variables and to analyse the prevalence of NCD risk factors. A heatmap or highlight table was used to visualise the relationship between NCD risk factors according to socio-demographic and economic characteristics. The colour scale used darker shades to indicate higher percentages and lighter shades to indicate lower percentages.

We employed multivariable logistic regression analysis to identify the factors associated with the NCD risk factors. Potential confounders, including age, sex, residence (urban/rural), education level, marital status, and regional variations, were adjusted for in the multiple logistic regression models. Adjusted odds ratios (AORs) were estimated using a logistic regression model to quantify the association between socio-demographic and economic factors and the likelihood of having risk factors. Regarding a heatmap table, the colour scale was a high AOR and significant P-value ≤0.05 coloured red and a low AOR and significant P-value ≤0.05 coloured green.

### Ethical considerations

We applied this protocol to the analysis of anonymised secondary data. Accordingly, informed consent was not required for this study. This study was approved by the Human Research Ethics Committee of the Faculty of Medicine, Prince of Songkla University (REC. 65-312-9-6).

## Results

### Characteristics of participants by type of non-communicable disease risk factor

Table 2 shows that the study included a weighted sample of 26,600,947 participants after excluding missing data. The majority of participants were female (57.00%), aged 45–59 years (32.09%), and resided in rural areas (53.00%). Most participants had primary education (46.46%) followed by secondary education (31.06%), and fewer participants had higher education (18.66%) or no education (3.81%). Most participants were living with a partner (61.31%); 20.62% were never married, and 18.08% were not living with a partner.

**Table 2.** Characteristics of the study population classified by socio-demographic and economic variables.

Characteristics	Column %	[95% CI]
**Sex**		
Male	43.00	[42.98–43.02]
Female	57.00	[56.98–57.02]
**Age (years)**		
15–19	2.86	[2.85–2.87]
20–24	5.59	[5.58–5.60]
25–44	31.41	[31.39–31.43]
45–59	32.09	[32.07–32.11]
60 or older	28.06	[28.04–28.08]
**Residence**		
Urban	47.00	[46.98–47.02]
Rural	53.00	[52.98–53.02]
**Education**		
No education	3.81	[3.80–3.82]
Primary	46.46	[46.44–46.48]
Secondary	31.06	[31.04–31.08]
Higher	18.66	[18.65–18.67]
**Living arrangement**		
Never married	20.62	[20.60–20.63]
Living with partner	61.31	[61.29–61.33]
Not living with partner	18.08	[18.07–18.09]
**Economic level**		
Very low	35.35	[35.33–35.37]
Low	14.39	[14.38–14.40]
Middle	18.70	[18.69–18.71]
High	18.04	[18.03–18.05]
Very high	13.53	[13.52–13.54]
**Thailand region**		
Bangkok	13.04	[13.03–13.05]
Central	30.60	[30.58–30.62]
Northern	17.74	[17.72–17.75]
North-eastern	26.14	[26.12–26.16]
Southern	12.49	[12.48–12.50]

The distribution of economic variables indicated that the largest proportion of participants were categorised into the very low economic level group (35.35%), followed by the middle economic level (18.70%), high economic level (18.04%), low economic level (14.39%), and very high economic level groups (13.53%).

### Prevalence of non-communicable disease risk factors

Table 3 shows the risk factors for NCDs categorised by socio-demographic and economic status. The most prevalent behavioural risk factor was an unhealthy diet (56.93%), followed by overweight or obesity (50.03%) and physical inactivity (42.70%). Alcohol consumption (29.73%) and smoking (16.61%) were the least prevalent. The prevalence of risk factors varied across subgroups, as detailed in Table 3. By economic level, the participants at the very low and low levels reported the lowest prevalence of alcohol consumption and following an unhealthy diet but the highest prevalence of physical inactivity (48.45%). In contrast, the prevalence of alcohol consumption and following an unhealthy diet increased at higher economic levels, while that of smoking decreased. Physical inactivity showed a U-shaped trend, with a higher prevalence in the subgroups with very low and very high economic levels.

**Table 3.** Prevalence of non-communicable disease risk factors (smoking, alcohol consumption, unhealthy diet, physical inactivity, overweight or obesity) classified by socio-demographic and economic characteristics.

Socio-demographic and economic variables	Total	Prevalence of non-communicable disease risk factors (%)				
		Smoking	Alcohol consumption	Unhealthy diet	Physical inactivity	Overweight/obesity
**Total**	**26,600,947**	**16.61**	**29.73**	**56.93**	**42.70**	**50.03**
**Sex**						
Male	11,460,169	36.34	51.01	61.70	42.30	51.44
Female	15,140,778	1.70	13.63	53.34	43.02	48.98
**Age (years)**						
15–19	760,444	9.93	20.03	54.00	58.30	23.74
20–24	1,485,779	18.09	38.51	52.40	44.33	32.10
25–44	8,354,337	19.12	38.70	61.48	39.80	48.65
45–59	8,535,406	18.13	32.37	60.57	37.61	57.27
60 or older	7,464,983	12.48	15.93	48.91	49.89	49.59
**Residence**						
Urban	12,502,580	15.33	30.77	57.71	42.12	50.23
Rural	14,098,368	17.77	28.82	56.26	43.24	49.87
**Education**						
No education	1,013,800	18.20	19.39	45.31	51.21	42.34
Primary	12,359,892	17.98	25.14	54.00	43.53	51.85
Secondary	8,263,186	18.32	36.47	59.72	41.62	49.43
Higher	4,964,070	10.06	32.07	62.02	40.77	48.11
**Living Arrangement**						
Never married	5,484,510	18.87	37.10	56.59	45.18	38.25
Living with partner	16,307,971	16.77	29.81	59.05	40.06	53.92
Not living with partner	4,808,467	13.53	21.07	50.18	48.90	50.34
**Economic level**						
Very low	9,403,717	12.40	18.16	48.66	48.45	47.52
Low	3,826,574	18.17	29.43	56.60	36.80	51.56
Middle	4,974,605	21.26	35.86	59.93	37.88	50.25
High	4,797,656	21.03	40.80	63.07	42.72	50.82
Very high	3,598,394	13.70	37.07	66.66	40.67	53.69
**Thailand region**						
Bangkok	3,468,317	17.02	30.10	63.96	30.45	50.33
Central	8,139,249	16.15	28.94	56.81	49.86	52.59
Northern	4,718,057	15.47	34.65	52.67	39.12	49.70
North-eastern	6,952,805	16.40	32.70	56.46	46.82	46.61
Southern	3,322,520	19.42	18.10	57.03	34.49	51.15

### Effects of socio-demographic and economic levels on non-communicable disease risk factors

**Figure 1.**
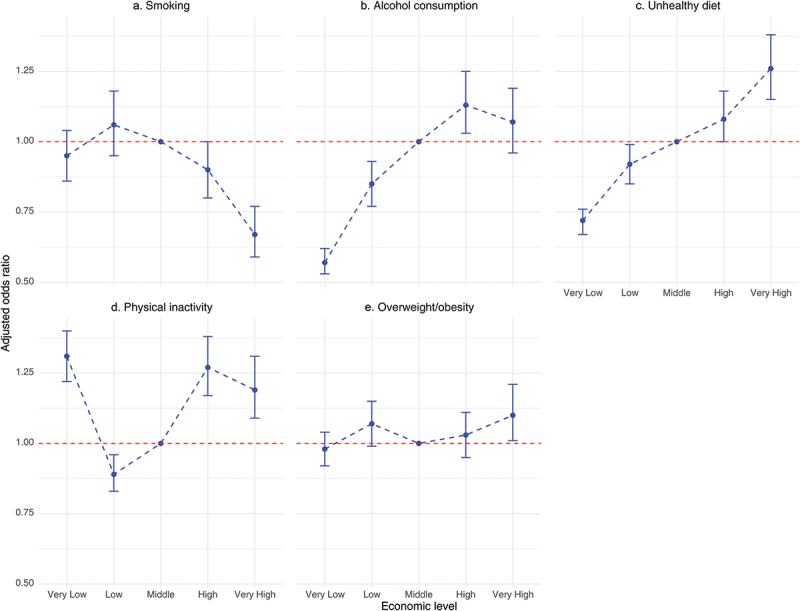
Adjusted odds ratios for behavioural risk factors of non-communicable diseases by economic level. Note: ‘middle’ economic level is the reference group for all comparisons.

The AORs revealed significant associations between socio-demographic and economic factors and health behaviours, as presented in Table 4 and Figure 1. The participants with very low and low economic levels had significantly lower odds for alcohol consumption and unhealthy dietary behaviours than those with a middle economic level (e.g. alcohol consumption: AOR = 0.57, 95% CI: 0.53–0.62 for very low; AOR = 0.85, 95% CI: 0.77–0.93 for low). Conversely, the participants with high and very high economic levels exhibited higher odds (e.g. alcohol consumption: AOR = 1.13, 95% CI: 1.03–1.25 for high; unhealthy diet: AOR = 1.26, 95% CI 1.15–1.38 for very high). These results demonstrated a dose–response relationship, with the odds progressively increasing from very low to very high economic levels. For smoking, the opposite pattern was observed. The participants with higher economic levels had slightly lower odds, with those with very high economic levels having the lowest odds (AOR = 0.67, 95% CI: 0.59–0.77). For physical inactivity, a U-shaped relationship was observed. The participants with very low economic levels had significantly higher odds (AOR = 1.31, 95% CI: 1.22–1.40) than those with middle economic levels, while those with low economic levels had lower odds (AOR = 0.89, 95% CI: 0.83–0.96). The participants with very high economic levels also showed increased odds (AOR = 1.19, 95% CI: 1.09–1.31), reflecting an elevated risk at both extremes of the economic spectrum. The participants with very high economic levels had slightly higher odds of overweight or obesity (AOR = 1.10, 95% CI: 1.01–1.21) than those with middle economic levels, while no significant differences were observed among the other economic level subgroups.

**Table 4.** Adjusted odds ratio showing the likelihood of the type of non-communicable diseases risk factors classified by socio-economic and demographic characteristics.

Variables	Adjusted odds ratio (95% CI)				
	Risk factor				
	Smoking	Alcohol consumption	Unhealthy diet	Physical inactivity	Overweight/obesity
**Sex**					
Male	Ref	Ref	Ref	Ref	Ref
Female	0.03 (0.02, 0.03)*	0.14 (0.13, 0.15)*	0.76 (0.73, 0.80)*	1 (0.96,1.05)	0.90 (0.86, 0.94)*
**Age (years)**					
15–19	Ref	Ref	Ref	Ref	Ref
20–24	2.83 (2.08, 3.86)*	2.84 (2.2, 3.67)*	0.79 (0.65, 0.95)*	0.63 (0.52, 0.76)*	1.39 (1.13, 1.71)*
25–44	3.45 (2.62, 4.54)*	3.03 (2.41, 3.81)*	0.97 (0.82, 1.13)	0.55 (0.47, 0.64)*	2.38 (1.98, 2.85)*
45–59	2.70 (2.04, 3.57)*	2.18 (1.73, 2.75)*	0.98 (0.83, 1.15)	0.51 (0.43, 0.60)*	3.24 (2.70, 3.90)*
60 or older	1.26 (0.95, 1.67)	0.78 (0.61, 0.98)*	0.71 (0.61, 0.84)*	0.77 (0.66, 0.91)*	2.49 (2.06, 3.00)*
**Residence**					
Urban	Ref	Ref	Ref	Ref	Ref
Rural	1.24 (1.15, 1.33)*	0.96 (0.90, 1.02)	1.11 (1.05, 1.16)*	0.90 (0.86, 0.95)*	0.97 (0.92, 1.01)
**Education**					
No education	Ref	Ref	Ref	Ref	Ref
Primary	0.87 (0.73, 1.04)	1.45 (1.23, 1.71)*	1.3 (1.17, 1.44)*	0.71 (0.64, 0.78)*	1.38 (1.25, 1.53)*
Secondary	0.61 (0.51, 0.73)*	1.63 (1.38, 1.93)*	1.38 (1.23, 1.55)*	0.70 (0.63, 0.79)*	1.51 (1.35, 1.69)*
Higher	0.32 (0.26, 0.39)*	1.22 (1.02, 1.46)*	1.35 (1.19, 1.53)*	0.71 (0.62, 0.80)*	1.28 (1.13, 1.44)*
**Living arrangement**					
Never married	Ref	Ref	Ref	Ref	Ref
Living with partner	0.99 (0.88, 1.10)	0.94 (0.86, 1.03)	1.24 (1.15, 1.33)*	0.84 (0.78, 0.91)*	1.53 (1.42, 1.65)*
Not living with partner	1.40 (1.22, 1.61)*	1.08 (0.97, 1.21)	1.06 (0.97, 1.16)	1.07 (0.98, 1.17)	1.39 (1.27, 1.51)*
**Economic level**					
Very low	0.95 (0.86, 1.04)	0.57 (0.53, 0.62)*	0.72 (0.67, 0.76)*	1.31 (1.22, 1.40)*	0.98 (0.92, 1.04)
Low	1.06 (0.95, 1.18)	0.85 (0.77, 0.93)*	0.92 (0.85, 0.99)*	0.89 (0.83, 0.96)*	1.07 (0.99, 1.15)
Middle	Ref	Ref	Ref	Ref	Ref
High	0.90 (0.80, 1.00)	1.13 (1.03, 1.25)*	1.08 (1.00, 1.18)	1.27 (1.17, 1.38)*	1.03 (0.95, 1.11)
Very high	0.67 (0.59, 0.77)*	1.07 (0.96, 1.19)	1.26 (1.15, 1.38)*	1.19 (1.09, 1.31)*	1.10 (1.01, 1.21)*
**Thailand region**					
Bangkok	Ref	Ref	Ref	Ref	Ref
Central	0.78 (0.67, 0.91)*	1.07 (0.94, 1.20)	0.74 (0.67, 0.82)*	2.43 (2.20, 2.69)*	1.10 (0.99, 1.21)
Northern	0.71 (0.61, 0.82)*	2.10 (1.86, 2.37)*	0.70 (0.64, 0.78)*	1.52 (1.38, 1.69)*	0.96 (0.87, 1.06)
North-eastern	0.82 (0.71, 0.96)*	2.02 (1.80, 2.28)*	0.83 (0.75, 0.91)*	2.15 (1.95, 2.38)*	0.83 (0.76, 0.92)*
Southern	1.13 (0.97, 1.31)	0.58 (0.51, 0.67)*	0.77 (0.70, 0.85)*	1.33 (1.20, 1.47)*	1.02 (0.93, 1.13)

*significant with P-value ≤ 0.05.

Figure 1 illustrates these trends, highlighting the dose–response relationship for alcohol consumption and an unhealthy diet, the inverse relationship for smoking, and the U-shaped relationship for physical inactivity.

## Discussion

### Principal findings and previous studies

A study on the prevalence of five behavioural risk factors for NCDs in the Thai population in 2021 revealed that behavioural risk factors varied according to economic status. The most prevalent risk factor was an unhealthy diet (56.93%), followed by overweight or obesity (50.03%), physical inactivity (42.70%), alcohol consumption (29.73%), and smoking (16.61%).

The prevalence of smoking (16.61%) was lower than the global average of 22.3%, most likely owing to effective tobacco control policies, including taxes and advertising bans, in Thailand [13]. The prevalence of alcohol consumption (29.73%) was also below the 2019 global average of 44% [14]. The highest prevalence of alcohol consumption is found in high-income regions, such as Australasia and Europe, and the lowest in predominantly Muslim regions, such as Northern Africa, the Middle East, and parts of South and Southeast Asia. Nevertheless, alcohol consumption in Thailand remains high compared to that in other Southeast Asian regions, and more regulatory intervention is necessary [14]. The prevalence of an unhealthy diet (56.93%) followed the global trends of high consumption of sodium, added sugars, and processed foods [15], while the level of physical inactivity (42.70%) was higher than the global average of 31%, which reflects the need for urgent intervention to increase active lifestyles in the face of urbanisation [16]. The prevalence of overweight and obesity (50.03%) was found to be slightly above the global average of 43%, reflecting an increasing burden of obesity due to diet and lifestyle transitions [17].

Our findings revealed that higher economic levels were associated with increased odds of alcohol consumption, unhealthy dietary behaviours, and overweight and obesity. These trends may be attributed to greater purchasing power in higher economic groups, which enables increased access to alcohol and high-calorie foods, as well as lifestyle preferences influenced by urbanisation and social norms. For instance, higher economic groups may experience greater exposure to alcohol marketing and dining cultures that promote high-calorie diets [18,19].

Conversely, an inverse relationship was observed with physical inactivity, where the lower economic strata demonstrated a higher prevalence, possibly owing to structural barriers such as limited access to recreational facilities, unsafe environments for physical activity, or occupational demands that limit time for exercise. For example, individuals in lower-income groups may engage in sedentary work environments or lack access to affordable gyms and safe outdoor spaces [20,21].

Smoking prevalence showed minimal variation across economic levels, with a slight decrease among higher economic groups. This pattern could be attributed to greater health awareness, better access to smoking cessation programmes, and higher tobacco taxation, reducing affordability for lower-income groups. However, cultural and peer influences may cause sustained smoking habits across economic strata [22,23].

### Strengths and limitations

This study has several strengths. First, we utilised a nationally representative data set, ensuring the generalisability of the findings to the Thai population. Second, the stratified sampling design and large sample size contribute to the reliability and robustness of the results. Lastly, the findings of this study present the details of the socio-demographic and economic factors that influence five major behavioural risk factors for NCDs, delivering insights that are valuable for shaping public health policies and interventions.

However, the study also has limitations. First, self-reported data on behavioural risk factors may be subject to recall bias or social desirability bias. Second, the cross-sectional study design prevents the establishment of causal relationships between economic levels and NCD risk factors. Lastly, in this study, we focused on individual NCD risk factors but did not explore the interactions or clustering of these factors, which could provide additional context to the findings.

### Implications and further studies

Our findings emphasise the complexity of the relationships between economic levels and health behaviours, reinforcing the need for targeted public health interventions. For higher economic groups, policy action should be aimed at reinforcing alcohol control measures by increasing taxes, having more stringent advertising bans, and launching public education campaigns to reduce excessive alcohol consumption. Front-of-pack nutrition labelling, additional taxes on sugary drinks, and more stringent advertising restrictions on unhealthy foods should also be supported to promote healthier diets, as defined in Thailand’s National NCD Prevention and Control Plan. For lower economic groups, priority should be given to improving access to physical activity by expanding public recreational spaces, such as walking and cycling paths in urban planning, and subsidising community fitness programs to counter sedentary behaviour. Further, smoking cessation programs need to be expanded in primary health care services, particularly in the universal health coverage system, to enhance accessibility and affordability.

Therefore, policies should address the specific needs of each economic group, promoting equitable access to resources that support healthier behaviours while mitigating the risks associated with economic transitions. Integrating these efforts into national health policies (e.g. Thailand’s Universal Health Coverage scheme, Thailand National Health Promotion Programs) and urban planning initiatives can help to address the socio-economic gradient in NCD risk factors more effectively. Greater intersectoral collaboration between organisations (e.g. the Ministry of Public Health, Thai Health Promotion Foundation, local governments) will enhance implementation and long-term sustainability. These intervention programs will address health inequalities by enabling all socio-economic groups to adopt healthier lifestyles and tackle the health consequences of Thailand’s economic transition.

Future studies should explore the mechanisms underlying these trends, particularly how socio-economic changes impact health behaviours over time. Longitudinal research is needed to confirm causal pathways and identify effective interventions tailored to diverse socio-economic contexts.

## Conclusion

Our findings highlight the prevalence and socio-demographic determinants of five key NCD risk factors in the Thai population. An unhealthy diet (56.93%), overweight or obesity (50.03%), and physical inactivity (42.70%) were the most common risk factors, with alcohol consumption (29.73%) and smoking (16.61%) being less prevalent. Higher economic levels were associated with increased odds of alcohol consumption, an unhealthy diet, and overweight or obesity, but a low prevalence of smoking. Physical inactivity showed a U-shaped relationship, with higher prevalence rates at both economic extremes.

Our findings emphasise the need for targeted interventions. Policies should be implemented to promote healthier diets and moderate alcohol consumption among higher economic groups and to improve access to physical activity for lower economic groups. Expanding smoking cessation programmes across all economic strata is a critical strategy. Future research should use longitudinal designs to confirm causal pathways and develop interventions to effectively address socio-economic disparities in NCD risk factors.

## Data Availability

The dataset was obtained with permission from Thailand’s National Statistical Office (https://www.nso.go.th/). Owing to ethical limitations governing the dissemination of a de-identified dataset, it is not permissible to share the data publicly. Interested researchers are welcome to contact the corresponding author, Polathep Vichitkunakorn (Polathep.v@psu.ac.th), to facilitate discussions regarding their requests for confidential data access. Furthermore, researchers can contact the National Statistical Office and Excise Department directly via services@nso.go.th to obtain the dataset.
